# An e-learning reproductive health module to support improved student learning and interaction: a prospective interventional study at a medical school in Egypt

**DOI:** 10.1186/1472-6920-12-11

**Published:** 2012-03-20

**Authors:** Rehab Abdelhai, Sahar Yassin, Mohamad F Ahmad, Uno GH Fors

**Affiliations:** 1 Department of Public Health, Cairo University, Cairo, Egypt; 2 National Tempus Office - Egypt, Giza, Egypt; 3 Department LIME, Karolinska Institutet, Stockholm, Sweden; 4 Department of Computer and Systems Science, Stockholm University, Stockholm, Sweden

**Keywords:** On-line learning, e-Learning, Reproductive health, Public health, Medical education, Egypt

## Abstract

**Background:**

The Public Health (PH) course at the medical college of Cairo University is based on traditional lectures. Large enrollment limits students' discussions and interactions with instructors.

**Aim:**

Evaluate students' learning outcomes as measured by improved knowledge acquisition and opinions of redesigning the Reproductive Health (RH) section of the PH course into e-learning and assessing e-course utilization.

**Methods:**

This prospective interventional study started with development of an e-learning course covering the RH section, with visual and interactive emphasis, to satisfy students' diverse learning styles. Two student groups participated in this study. The first group received traditional lecturing, while the second volunteered to enroll in the e-learning course, taking online course quizzes. Both groups answered knowledge and course evaluation questionnaires and were invited to group discussions. Additionally, the first group answered another questionnaire about reasons for non-participation.

**Results:**

Students participating in the e-learning course showed significantly better results, than those receiving traditional tutoring. Students who originally shunned the e-course expressed eagerness to access the course before the end of the academic year. Overall, students using the redesigned e-course reported better learning experiences.

**Conclusions:**

An online course with interactivities and interaction, can overcome many educational drawbacks of large enrolment classes, enhance student's learning and complement pit-falls of large enrollment traditional tutoring.

## Background

Research into teaching methodologies indicates that assigning a good tutor to individual students can produce the most effective learning results. Over 20 years ago, Bloom demonstrated that students who receive one-on-one instruction performed two standard deviations better than students in traditional classrooms [1]. The positive learning outcomes of one-on-one tutoring have been credited to students' participation in discussions and tutorial dialogues [2]. In addition, a skilled tutor can directly assess the student's strengths and weaknesses and tailor the presentation of instructional material to the learner's needs [3,4]. However, finding a skilled tutor for each student is, in most countries, unrealistic and economically not feasible.

It is usually assumed that a low student-faculty ratio is a necessity for quality education. However, in many colleges worldwide and especially in introductory courses, enrollments are usually high. The predominant form of instruction in such high enrollment courses is large lectures as it is assumed that these are the only low-cost alternatives available. Such lectures have been criticized for treating all students as identical individuals, ignoring the fact that students have different levels of interest, learning styles, motivation, attention span and ability to learn [5]. For example, while students with weaker skills need more attention and opportunities for interaction, students with stronger skills need opportunities to excel and accelerate their learning independently [6]. Additionally, current students, who are dubbed as the "net-generation", seem to have less attention span than previous generations [7]. This makes it more difficult for even a very professional lecturer to maintain students' attention in a large lecture presentation setting. In summary, traditional large lecture presentations have problems in engaging the students and in meeting their individual learning styles.

To give students a better opportunity for interaction in large enrollment classes, some universities combine large lecture presentations with discussion sections in smaller groups. However this multiple sections model also has its challenges. Sometimes these sections are often still quite large and are dominated by the same presentation techniques. Additionally, the instructor may develop his or her own set of course materials and deliver what is basically the same material in his or her own style. Therefore, although in theory this model may lead to better interaction with students, in practice this model often produces a remarkable lack of uniformity in learning outcomes due to lack of coordination and inconsistency [6].

The advances in information and communication technology have directed attention to online learning methods, also known as e-learning. With the introduction of the Internet and the World Wide Web, e-learning has shown a major potential for providing more flexible access to courses content and instructions. Therefore, e-learning might address large enrollment problems by delivering course contents and instructions to learners anytime and anywhere. Furthermore, well designed e-learning courses can enhance the quality of learning experiences and outcomes by promoting the concept of learner-centered courses and encouraging students self-learning. A prevailing presumption is that learning a complex body of knowledge effectively, requires a community of learners that can be expanded and supported through e-learning [8,9]. Another conjecture is that asynchronous discourse is inherently self-reflective and therefore more conducive to deep learning, than is synchronous discourse [10,11].

### The actual challenge

In 2004, the Egyptian Supreme Council of Universities evoked an initiative for reforming the undergraduate medical education. The vision of the Egyptian Medical schools was set to produce competent graduates who should be able to perform their jobs according to the accepted international standards [12]. In response, the Department of Public Health and Community Medicine (DOPHCM) at the Faculty of Medicine, Cairo University sought means to support such a vision. The department is responsible for teaching the Public Health (PH) course which is a required course in the Egyptian medical schools' curricula. The challenge was to enhance the learning outcomes for this course when enrollment, approximates about 1500 students per academic year.

This course runs for eight weeks, and is offered four times every academic year (in four rounds) to accommodate the 1500 students. Therefore, the enrollment in each offering exceeds 350 students. Medical students enroll in this course during their fourth year of college (out of 6 years in total). Seven two-hour lectures are offered every week in large lecture halls to accommodate the students. In addition, the 350 students are divided into 10 groups of about 35 students each. Each group attends about eight tutorial classes and two site visits (two-hours each) per week. However, even with this set-up, the previously mentioned lack of uniformity in learning outcomes [6] is a major concern as the rather large group size is also still limiting student-to-teacher interactions.

### Study objective

The objective of the study was to determine whether the undergraduate medical students' course that is taught traditionally (in a face to face setting), could also be taught in a complementary distance learning setting. We hypothesized that a supplementary distance learning alternative would be at least equal to the traditional format in developing student knowledge, skills and attitudes.

Our intention was to demonstrate that constructing a complementary distance e-learning course which integrates technology into learning can produce learning results that exceed the results obtained by large enrollment lecture format alone. Student-faculty interaction is an important component of the instructional format which can vanish in large enrollment classes. Therefore, in this study, alternative forms of interactions were sought in order to raise students' achievements.

## Methods

### Developing the e-learning module

The Reproductive Health (RH) part of the PH course constitutes about 15% of the curriculum and is four weeks long. It includes sections on Maternal and Child Health, the Family planning program in Egypt, as well as indicators to measure health. In the traditional setting this part is given in 7 grand lectures, 8 tutorials and 4 site visits (in total 38 hours of teaching). Additionally, the students are instructed to read the comprehensive text book on the subject.

The open source Course Management System (CMS) "Moodle" was utilized to develop an e-Learning course covering the RH part of PH curriculum. The e-Learning course contains 4 modules: Measurement of health (10 lessons), Maternal health program (17 lessons), the Family planning program (4 lessons), and Child health care (14 lessons). Each lesson is estimated to take one hour each and is followed by a lesson quiz with multiple choice questions (MCQ) and extended matching questions. All e-Learning material was based on the required course textbook and the intended learning outcomes (ILO's). However, to facilitate learning the original material was complemented by new visuals, animations and other interactive materials. The Course Management System environment also offers student-student as well as student-teacher communication via a chat room (open 24 hours), forum discussions, and the possibility to send messages between the users as well as faculty members.

Deadlines for completion of each module were set by the course facilitators. Completion of the course took on average five weeks. Course facilitators (two senior and one junior staff member) spent approximately 20-25 hours a week participating with all students in online discussion forums and answering questions.

### E-learning course assessment

Each lesson of the e-Learning course is followed by a number of MCQ and matching questions with a 70% passing grade requirement to move on to the next lesson. Additionally, each module of the e-Learning course starts with a pre-quiz and ends with a post-quiz. Quizzes include 20 MCQ and matching questions, which appear at random, from all lessons of the module. Students are allowed one attempt for the pre-quiz and five attempts for the post-quiz. The final score for the module is based on the average of the five post-quizzes.

Polls about the different sections were developed to assess satisfaction with the module content and instructional design. Additionally, behavior/attitude-based assessments were also done based on contributions to the on-line discussion forums. A final questionnaire was developed to assess attitudes and perceptions towards e-learning in comparison to the traditional lectures.

### Study design and participants

This prospective interventional study included two student groups. "Group 1" includes students who volunteered to use the e-Learning course. This group was assessed by online post-modules' quizzes as mentioned above and a final online exam. "Group 2" were those who received the traditional lecturing approach only. The whole class (including both groups) was a highly motivated group of students whose grades, according to university records, were high. Initial paper instructions were given to the first group of students on how to use the on-line system, methods of assessments and deadlines.

The study used a prospective design, were students attending the DOPHCM round were invited at the 1^st ^week of the round to the e-learning module on a voluntary basis. Over three rounds a total of 171 students participated in the e-learning module.

The students enrolled in the e-Learning course were also invited (by email and phone) to three focus group discussions. In the focus groups, the students discussed what they enjoyed most and if they found any limitations or difficulties during the course. The students who did not volunteer to use the system were also invited (by email and phone from their peers using the e-learning system) to similar group discussions where they gave insights to the reasons of non-participation.

At the end of each round, a random sample of students from group 1 and group 2, were asked to complete two questionnaires; the first was the RH knowledge questionnaire, and the second a Course Experience Questionnaire (CEQ) adapted from *Ramsden 1991 *and *Ramsden 2003 *[13,14]. A total of 295 students completed the RH questionnaire (140 taking the e-Learning course and 155 non-participants in the e-Learning course) and 200 students completed the CEQ (97 from the e-Learning course and 103 non-participants in the e-Learning course). A third questionnaire was introduced to 106 students who did not use the e-learning course to detect reasons for non-participation.

### Learning assessments

1- Students participating in the e-learning module were assessed by the pre and post quizzes as well as by the final online quiz. Online quizzes consisted of a range of MCQ and extended matching questions, covering the sections addressed in each module. All quizzes were timed and since questions appeared randomly for each student, it was not possible for students to share answers.

2- The CEQ reflects students' opinion concerning the learning process from students' perspectives.

3- The RH knowledge questionnaire was a paper based questionnaire introduced to students during their final week of the PH course. Students were asked to fill it anonymously indicating if they participated in the e-learning course or not.

The RH knowledge questionnaire and online quizzes were specifically developed to cover the intended learning outcomes of all areas of the RH part of the PH course. Content validity was determined by a senior member of the PH staff. Then the final e-learning course was pilot-tested before allowing the volunteering students to register in the course.

### Data manipulation, scoring, and analysis

Questions on the CEQ were grouped into five categories, namely;

• ***The Good Teaching Scale (GTS) ***which contains 6 questions covering practices such as giving feedback to the students on their progress, explaining things, and making the course interesting.

• ***The Clear Goals and Standards Scale (CGS)***, contains 4 questions and measures the clarity of course goals and the expected standards of students attending the course.

• ***The Appropriate Assessment Scale (AAS) ***contains 3 statements that attempt to measure the students' perceptions of the course assessments.

• ***The Appropriate Workload Scale (AWS)***, which contains 4 questions aimed to measure the student's perception of the amount of work needed for the course.

• ***The Generic Skills Scale (GSS) ***containing 6 questions takes into account the extent to which the course adds to the generic skills of the students e.g. decision and problem solving skills and capacity for self learning.

All questions were rated on a 5 point Likert scale from strongly disagree with a score of 1 to strongly agree with a score of 5. Scores for each scale were added and mean scores were reported

Questions on the RH knowledge questionnaire were scored as incorrect or correct and a final total score was calculated. At the end of the RH questionnaire, students were asked to evaluate the ease of the questions on a 3 point Likert scale (easy, moderate and difficult).

The Statistical Program for Social Sciences (SPSS) version 15 was used for data analysis. Analysis included simple frequencies and descriptive analysis (Mean and Standard Deviations). Statistical tests of significance used were the Chi Square test, the Paired *t*-test and the Student's *t*-test, as appropriate. A P - value < 0.05 was considered significant.

### The focus groups

All meetings were recorded and transcribed manually. After transcription, responses from each meeting were subjected to content analysis which permitted category or theme creation based on frequency of participants' responses.

### Ethical considerations

The study was approved by the Public Health Department Council, Faculty of Medicine - Cairo University. All assessment questionnaires were collected in an anonymous and voluntary manner. Data of students participating in the e-learning course was preserved confidentially throughout the study in accordance with the Declaration of Helsinki.

## Results

Of the 171 students enrolled in the e-learning module, the majority were females (68% - n = 116) and 32% were males (n = 55). On the other hand, students in group 2 had a nearly equal distribution, with males representing 48% (n = 75) and females 52% (n = 80). All Students' age ranged from 20 to 23 years with mean age of 20.4 ± 0.6 years. The learning outcomes for group 1, as measured by improved course scores between the pre- and post-quizzes were highly significant with P < 0.001 [Table 1].

**Table 1:** Comparison of students mean quiz scores, before and after taking the e-learning lessons, by module

Module Quiz Scores		Mean ± SD	Paired difference ± SD	95% CI	Paired t -test P - value
**Measurements of Health**	***Post***	86.93 ± 16.69	28.91 ± 25.43	24.78-33.05	< 0.001
	***Pre***	58.02 ± 20.86			

**Maternal Health**	***Post***	90.47 ± 14.67	23.63 ± 19.97	19.72-27.52	< 0.001
	***Pre***	66.84 ± 16.52			

**Family Planning**	***Post***	96.04 ± 10.18	24.49 ± 22.24	20.29-28.70	< 0.001
	***Pre***	71.55 ± 21.62			

**Child Health**	***Post***	93.47 ± 10.02	25.45 ± 18.97	21.63-29.28	< 0.001
	***Pre***	68.02 ± 17.62			

Number of students = 171. SD = Standard deviation; and 95%CI = 95% confidence interval

When comparing mean RH knowledge scores between students participating in the e-learning module (n = 140) and those attending the regular lectures only (n = 155), we found a highly statistical significant improvement for students in the e-learning group (P < 0.001). No difference was found among males and females attending the e-learning module, regarding their scores (P = 0.48). On the other hand, females attending the regular lectures had slightly higher mean knowledge scores than males and this finding was border line significant [Table 2].

**Table 2:** Reproductive health knowledge scores among participating students

Study groups		Mean ± SD	95% CI	Student's t -test P - value
**All Students**	**E - learning (n = 140)**	15.40 ± 2.46	-4.18 -- - 2.66	< 0.001
			
	**Non e - learning (n = 155)**	11.97 ± 2.78		

**E - learning**	**Males (n = 64)**	15.09 ± 2.33	-1.72 -- 0.82	0.48
			
	**Females (n = 76)**	15.54 ± 2.54		

**Non e-learning**11.52 ± 2.97	**Males (n = 75)**	11.52 ± 2.97	-1.75 -- -0.005	0.049
			
	**Females (n = 80)**	12.40 ± 2.53		

SD = Standard deviation; and 95%CI = 95% confidence interval

We explored students' perceptions towards the ease of questions on the knowledge questionnaire. We found that nearly a quarter of students in the e-learning group (24.3% - n = 17/70) reported they found the questions easy versus only 7.7% of students in the traditional teaching group (n = 9/117). Inversely, 43.6% of students attending the traditional lectures (n = 51/117) found questions difficult versus only 5.7% of students from the e-learning group (n = 4/70), and these findings were highly significant with P < 0.001 [Table 3].

**Table 3:** Perceptions of students towards the reproductive health knowledge questionnaire

**Study Group**	**Reproductive health knowledge questions**			**Total** **No^†^. (%)**	**χ^2^** **P - value**
			
	**Easy** **No. (%)**	**Moderate** **No. (%)**	**Difficult** **No. (%)**		
	
**E - Learning**	17 (24.3)	49 (70.0)	4 (5.70)	70 (100.0)	< 0.001
**Non e - learning**	9 (7.70)	57 (48.7)	51 (43.6)	117 (100.0)	

No^† ^= not all students (from both groups) answered this question i.e. presence of missing valuesχ^2 ^= Chi Square test

At the end of PH round, another random sample of students were asked to complete the CEQ. Both study groups filled out the questionnaire. A comparison of mean scores on the CEQ scales was conducted between both groups of the study. Students enrolled in the e-Learning course had higher significant mean scores for the Good Teaching Score. Similarly, they had significantly higher scores for the Clear Goals Scale and the Appropriate Workload Scale. On the other hand, no difference between both groups was detected for the Appropriate Assessment Scale and the Generic Skills Scale [Table 4].

**Table 4:** Comparison of course experience questionnaire scores among participating students

CEQ Scales	E-learning^†^	Mean ± SD	95% CI	Student's t -test P - value
**Good Teaching Scale**	**Yes**	21.35 ± 5.36	- 3.39 - - 0.02	0.035
			
	**No**	19.67 ± 4.47		

**Clear Goals & Standards**	**Yes**	13.65 ± 3.28	-3.08 - - 0.95	0.043
			
	**No**	11.98 ± 2.74		

**Appropriate Assessment**	**Yes**	9.29 ± 2.39	- 0.93 - 0.82	0.893
			
	**No**	9.23 ± 2.53		

**Appropriate workload**	**Yes**	14.88 ± 2.38	- 2.54 - - 0.59	0.002
			
	**No**	13.30 ± 2.96		

**Generic Skills Scale**	**Yes**	21.82 ± 4.32	- 0.18 - 1.34	0.77
			
	**No**	21.59 ± 4.52		

E-learning^† ^Yes (n = 97) No (n = 103)

SD = Standard deviation

95%CI = 95% confidence interval

We identified causes why students did not participate in the e-learning course by analyzing the third questionnaire's data. The main reasons claimed were mainly due to commuting daily from governorates outside Cairo and the extensive amount of required course material to study (38.7% of responses each - n = 41/106). About a fifth (19.8% - n = 21/106) lived in the university dorm with limited computer and internet facilities while about 22% (n = 23/106) reported inability to access the internet. About one third (32.1% - n = 43/106) reported having initially enrolled in the e-learning, but missed the first deadline for completion of the first module, and nearly 19% could not keep up with the deadlines (n = 20/106), and were not able to continue the whole module. Another 13% (n = 14/106) of students reported *"not liking" *deadlines (like the ones in the e-learning modules). Only 12% of surveyed students reported that they considered e-learning *"a waste of time" *(n = 13/106). However, nearly 62% (n = 65/106) expressed their wish to participate in the e-learning module in the future [Table 5].

**Table 5:** Causes mentioned for non-participation in the e-learning course

Causes for non-participation^†^	Frequency **No**.	Percent
**Lack of time due to travel from outside Cairo to the university**	41	38.7
**Lack of time due to extensive studies**	41	38.7
**No access to computer/internet**	23	21.7
**Lives in University Dorm with limited facilities**	21	19.8
**Missed the deadline for the first module**	34	32.1
**Could not keep up with the deadlines**	20	18.9
**Don't like deadlines**	14	13.2
**Considers e-learning a waste of time**	13	12.3
**Future desire to participate in e-learning**	65	61.9
**Total^†^**	**106**	**100.0**

^†^Multiple answers were allowed

Both groups of students were invited to three group discussions. Analysis of focus group discussions data yielded three major themes. First, students enrolled in the e-learning course highlighted how the course helped improve their outcome of learning. Students reported that ***"they achieved better through the e-learning course"***, and that e-learning ***"helped plan their studies and helped (them) to understand the subject"***. Students raised the issue of their wish for the e-learning course to include other parts of the PH curriculum other than RH to help them with their studies for the whole course, and if possible to have sections in Arabic (as the e-learning modules were written in English). The second theme focused on interaction. Students were very positive regarding interaction, and feedback from the tutors responsible for the e-learning course and from each other in the forums. They expressed that they ***"could now communicate between each other and with the teachers using the e-learning course platform"***. They also described how the e-learning course ***"helped them with learning through interactions with each other and how they were motivated to create links to new learning material"***. They expressed a wish for more images and interactive animations and videos as well as links to external resources, although they had created some of their own that were approved by the course facilitators. The third theme was related to the e-course system. Students expressed they had a ***"Very Good experience" ***with the e-learning system, and raised the issue of their wish for ***"the system to re-open after deadlines" ***to give them a chance to revise for exams.

Analysis of focus group discussions data for students not enrolled in the e-course generated two themes. The first addressed issues related to the course. They highlighted the fact that the e-course was time consuming and required commitment to course deadlines. Students expressed that ***"it consumed too much time"***, that they ***"ran out of time" ***and ***"could not keep up with the course deadlines"***. The second theme focused on the access to computers and internet. Most of them had no personal access to internet and/or computers. Additionally, the university does not provide any computer and internet connection facilities for students. However, they expressed wishes to access the e-Learning course before the end of the academic year through cyber cafés for exam revisions.

Results of the final online questionnaire showed that students' satisfaction with the e-learning course exceeded 95% (163/171) and that the majority (92% - n = 157/171) agreed that the course was well organized and ran smoothly. They expressed their appreciation to have the "***chance to do all they wanted and needed to do freely, whenever they wanted"***. Their opinions on traditional lectures were that these were "***not interactive" ***and that they were "***passive listeners only with little chance to participate"***.

## Discussion

Thanks to the Internet and steady growth of educational technologies, the number of e-learning resources available to educators has dramatically increased. Repositories or digital libraries have been established within medical education, to manage access to e-learning materials [15]. In undergraduate medical education, e-learning offers learners materials for self-instruction and collaborative learning. The idea of a collaborative learning community is a critical concept in medicine where high order learning is desired [5]. Although some institutions have tried to use e-learning as a stand-alone solution to updating or expanding their curricula, it is best believed to begin with an integrated strategy that considers the benefits and burdens of e-learning before revising the curriculum [16]. Additionally, there is evidence for the effectiveness and acceptance of e-learning within the medical education community [17], especially when combined with traditional teacher led activities [18], which coincides with our methodology.

As with other educational materials, there are two major approaches to the evaluation of e-learning: process and outcomes. In our study we concentrated on evaluation of the outcome through measuring changes in learners' knowledge, or attitudes. The evaluation framework outlined by Kirkpatrick, can be used to evaluate e-learning interventions. The Kirkpatrick model defines four levels of evaluation based on outcome: satisfaction, learning, change in learner behavior, and organizational change/patient outcome [15], all of which coincide with our study except the fourth item. Our findings show that despite that e-learning was a new thing for the students they were highly satisfied with the e-learning course. The majority enjoyed learning using this method. Their overall scores improved as they proceeded in the e-learning module. All differences between pre and post-quiz scores of the e-learning module were highly significant. These findings are similar to those of other studies evaluating online courses [19]. When comparing reproductive knowledge scores between students participating in the e-learning course and those attending the regular lectures only; we found a highly statistical significant improvement for students in the first group. This coincides with other findings that show effect sizes favored e-learning compared to classroom instruction [20]. No difference was found among males and females attending the e-learning course, regarding their scores which shows no sex preference concerning e-learning.

We explored students' perceptions towards the ease of questions on the knowledge questionnaire. We found that nearly a quarter of students (24.3%) enrolled in the e-course (group 1) reported they found the questions easy. Inversely, 44% of students attending the Face-to Face traditional lectures (group 2) found questions difficult which may be attributed to better learning behavior for group 1.

A comparison of mean scores on the CEQ scales was conducted between both groups of the study. Students enrolled in the blended-learning course had higher significant mean scores for the Good Teaching Scale. This is consistent with another study that reported instructor ratings to be strongly correlated with student satisfaction [21]. Similarly, students on the e-learning course had significantly higher scores for the Clear Goals Scale and the Appropriate Workload Scale which agree with reflections of ***Norman 2008 ***showing e-learning to be more efficient than its alternatives where students participating in e-learning are reported to achieve the same amount of work in about 30% less time [22]. On the other hand, no difference between both groups was detected for the Appropriate Assessment Scale and the Generic Skills Scale which partly concur with a study that found no significant differences between student groups on achieving team process skill [18]. An observation of a more positive achievement regarding course learning objectives, perceived by students in the first group, is consistent with our findings. Researchers concluded that the results provide evidence to support e-learning format without compromising pedagogy. They also suggest that this format enhances students' perceptions of their learning [18].

The results of our focus group discussions showed that students were satisfied with the e-system and expressed they had a "***very good experience with the e-learning system"***. Additionally, improved learning outcome was stressed as the e-course **"*helped plan their studies" ***and **"*helped them understand the subject"***. They especially enjoyed the "***chance to do all they wanted and needed to do freely, whenever they wanted" ***and how ***"the e-learning course helped them with their learning"***. These findings iterate those reported in 2007 about the flexibility of e-learning [23], allowing learners' greater control over the learning environment and freedom to move at their own pace, thus adapting to characteristics of individual learners such as cognitive and learning styles.

### Limitations of the study

The students in the e-learning group were not prevented from attending the traditional lectures as well, which means that they might have done so. However, when asked, the majority of students in the e-learning course did not attend the lectures to a major degree. Although they volunteered for the course, their very superior learning outcomes as compared to the "traditional group" clearly indicated that the e-learning course was the main reason for their better results. This is because there was a highly motivated group of students whose grades, according to university records, were high yet did not choose to become involved in the e-course. Hence, the detected difference can be attributed to the e-course. Another limitation is that it was not possible to assess retention of knowledge, since students in each round move on to other departments and it was not possible to reach them at a later date. Furthermore, although the analysis used for the focus group data was a simple one, it was considered sufficient for the purpose of this research.

## Conclusions

Our results denote that an online course with activities for interaction between students and instructors and amongst students can overcome many educational drawbacks encountered in large enrolment classes, enhance student's learning thus overcoming pit-falls of traditional learning. In addition, student's perceptions of diversified visual and interactive e-learning content elements were very positive. The integration of e-learning into undergraduate, graduate, and continuing medical education will promote a shift toward adult learning in medical education, wherein teachers no longer serve solely as distributors of content, but become facilitators of learning and assessors of competency. It is recommended that universities with large enrollment classes adopt e-learning and provide facilities for its implementation. This can be achieved by providing computer halls with internet connections on campus and at university dorms.

## Abbreviations

AAS: Appropriate assessment scale; AWS: Appropriate workload scale; CEQ: Course experience questionnaire; CGS: Clear goals and standards; CMS: Course management system; DOPHCM: Department of public health and community medicine; GSS: Generic skills scale; GTS: Good teaching scale; ILO: Intended learning outcomes; MCQ: Multiple choice questions; PH: Public health; RH: Reproductive health; SPSS: Statistical program for social sciences.

## Competing interests

The authors declare that they have no competing interests.

## Authors' contributions

RA contributed to conceptualization and study design, development of study materials and tools, designing the e-course, study implementation, data acquirement, analysis & interpretation. She participated in drafting, reviewing and gave final approval of the manuscript. SY helped conceptualize the focus of the study, development of study materials and tools, data analysis & interpretation. She also helped in drafting, reviewing and gave final approval of the manuscript. MFA contributed to the concept and design of the study, with major inputs in designing the e-learning course and its implementation within the CMS. He also helped in drafting, reviewing and approval of the manuscript. UGHF helped conceptualize the study design, and development of tools. He also shared in interpretation of data. He critically reviewed and gave final approval of the manuscript. All authors read and approved the final manuscript.

## Pre-publication history

The pre-publication history for this paper can be accessed here:

http://www.biomedcentral.com/1472-6920/12/11/prepub
